# Prevention of mother-to-child transmission of human immunodeficiency virus among pregnant women using injecting drugs in Ukraine, 2000–10

**DOI:** 10.1111/j.1360-0443.2011.03609.x

**Published:** 2012-01

**Authors:** Claire Thorne, Igor Semenenko, Ruslan Malyuta, for the Ukraine European Collaborative Study Group in EuroCoord

**Affiliations:** 1 MRC Centre of Epidemiology for Child Health, UCL Institute of Child Health, University College LondonLondon, UK; 2 Perinatal Prevention of AIDS InitiativeOdessa, Ukraine

**Keywords:** HIV, injecting drugs, mother-to-child, pregnancy, pregnancy outcomes, prevention, transmission

## Abstract

**Aims:**

To compare clinical status, mother-to-child transmission (MTCT) rates, use of prevention of (PMTCT) interventions and pregnancy outcomes between HIV-infected injecting drug users (IDUs) and non-IDUs.

**Design and setting:**

Prospective cohort study conducted in seven human immunodeficiency virus/acquired immune deficiency syndrome (HIV/AIDS) Centres in Ukraine, 2000–10.

**Participants:**

Pregnant HIV-infected women, identified before/during pregnancy or intrapartum, and their live-born infants (*n* = 6200); 1028 women followed post-partum.

**Measurements:**

Maternal and delivery characteristics, PMTCT prophylaxis, MTCT rates, preterm delivery (PTD) and low birth weight (LBW).

**Findings:**

Of 6200 women, 1111 (18%) reported current/previous IDU. The proportion of IDUs diagnosed with HIV before conception increased from 31% in 2000/01 to 60% in 2008/09 (*P* < 0.01). Among women with undiagnosed HIV at conception, 20% of IDUs were diagnosed intrapartum versus 4% of non-IDUs (*P* < 0.01). At enrolment, 14% of IDUs had severe/advanced HIV symptoms versus 6% of non-IDUs (*P* < 0.001). IDUs had higher rates of PTD and LBW infants than non-IDUs, respectively, 16% versus 7% and 22% versus 10% (*P* < 0.001). IDUs were more likely to receive no neonatal or intrapartum PMTCT prophylaxis compared with non-IDUs (OR 2.81, p < 0.001). MTCT rates were 10.8% in IDUs versus 5.9% in non-IDUs; IDUs had increased MTCT risk (adjusted odds ratio 1.32, *P* = 0.049). Fewer IDUs with treatment indications received HAART compared with non-IDUs (58% versus 68%, *P* = 0.03).

**Conclusions:**

Pregnant human immunodeficiency virus-infected injecting drug users in Ukraine have worse clinical status, poorer access to prevention of mother-to-child transmission prophylaxis and highly active antiretroviral therapy, more adverse pregnancy outcomes and higher risk of mother-to-child transmission than non-injecting drug user women.

## INTRODUCTION

Injecting drug use (IDU) accounts for one in three new human immunodeficiency virus (HIV) infections outside subSaharan Africa [1], and drives the escalating HIV epidemic in eastern Europe and Central Asia (EE&CA). In Ukraine, an estimated 1–2% of the 45.8 million population are injecting drug users (IDUs) [2], and the country has the second largest population of opioid users in EE&CA [3], although increases in stimulant injecting have been reported [4,5]. Ukraine has the highest HIV prevalence in Europe, at 1.63% in 2007 [4]. Sharing of injecting equipment, front- and back-loading and purchase of syringes pre-filled with home-made opiates are believed to have contributed to the explosive spread of HIV among IDUs [5–7], with HIV prevalences of 23–70% reported in sentinel surveys in cities including Kiev and Odessa [7–10]. Approximately 25% of IDUs in Ukraine are female [8,11]. Female IDUs have an elevated HIV risk compared with males [7,8], reflecting more risky injecting practices, including being injected by others [12,13], and sexual risks, particularly multiple partners (many also IDUs) and sex work [6,8,13–16].

Low uptake of health services, including antenatal care (ANC), is a well-recognized problem among female IDUs [17,18], including those with HIV [19]. In addition to the risk of neonatal abstinence syndrome (NAS), infants of IDUs may be at increased risk of preterm delivery (PTD), intrauterine growth retardation and low birth weight (LBW) as well as exposure to maternal infections such as HIV, sexually transmitted infections (STI), hepatitis B (HBV) and C virus (HCV) [17,20–22]. In Ukraine, ANC services are provided free of charge, with more than 90% coverage. Infant mortality was 13 per 1000 live births in 2009 [23]. Routine antenatal HIV screening was established in Ukraine in 2000: testing takes place at pregnancy registration, and for women testing negative repeat testing occurs at approximately 30 weeks gestation. Rapid intrapartum testing for women without ANC testing was introduced nationally in 2003. HIV/AIDS treatment and care services in Ukraine are organized through a network of regional HIV/AIDS Centres. Substantial progress has been made in reducing rates of mother-to-child transmission (MTCT) of HIV in Ukraine [24]. Little is known about access of IDUs to prevention of MTCT (PMTCT) services or their pregnancy outcomes, including MTCT, in this setting, although limited data from western Europe and the Russian Federation have demonstrated HIV-infected IDUs to be at increased risk of non-receipt of PMTCT prophylaxis [25–27] and antenatal care [19].

Our aim was to compare clinical status, MTCT rates and risks, use of PMTCT interventions and pregnancy outcomes between HIV-infected IDUs and non-IDUs participating in a prospective cohort study of pregnant HIV-infected women and their children in Ukraine.

## METHODS

The European Collaborative Study (ECS) is an ongoing prospective cohort study established in 1985 in western Europe, with centres from Ukraine joining in 2000 [28]. The ECS was established to estimate the rate of and risk factors for MTCT and to investigate the natural history of vertically acquired HIV infection. Over the intervening years the objectives have been refined, consistent with changing management of HIV infection in pregnancy and the developing HIV epidemic in Europe, with a current focus on uptake and safety of PMTCT interventions. The ECS is part of EuroCoord, a European network of HIV/acquired immune deficiency syndrome (AIDS) cohort studies (http://www.eurocoord.net).

Women identified as HIV-infected before or during pregnancy, or through intrapartum testing, and delivering a live-born infant, are eligible to enrol with informed consent. Seven HIV/AIDS centres in Ukraine participate: Odessa, Mykolaiv and Simferopol (since January 2000), Kyiv, Donetsk and Mariupol (since September 2006) [24] and Kriviy Rig (since September 2008). Data were collected anonymously on study-specific questionnaires, using study serial numbers without personal identifiers (linked anonymous data). Information collected included maternal socio-demographic characteristics (date and country of birth, age at leaving full-time education, marital status, ethnicity, history of IDU, timing of last IDU), HIV-related data [timing of HIV diagnosis, World Health Organization (WHO) clinical stage, CD4 count, antenatal antiretroviral use] and delivery and infant characteristics (date and mode of delivery, birth weight, head circumference, gestational age, use of intrapartum and neonatal PMTCT prophylaxis, presence of NAS, other perinatal problems, HIV infection status). After delivery, infants are followed-up to establish infection status, with infected children then followed-up 6-monthly [29]. There is no maternal follow-up after delivery.

A nested substudy of the ECS was established in December 2007, with the aim of obtaining longitudinal information on childbearing HIV-infected women after delivery in order to investigate the impact of treatment, coinfection and exposure to abbreviated antenatal PMTCT prophylaxis on prognostic markers of HIV disease progression. Five sites of the ECS participate (Odessa, Kyiv, Donetsk, Mykolaiv and Kriviy Rig) [30], collecting initial postnatal data approximately 3–6 months after delivery and follow-up data annually thereafter. With regard to the ECS, data are linked anonymously. At enrolment, one questionnaire is completed by the woman and a clinical questionnaire is completed by her physician. Variables collected include additional information on pregnancy not available in the ECS (receipt of ANC, whether or not the pregnancy was planned), socio-demographic variables (accommodation type, partner's HIV infection status, imprisonment history), current alcohol use, ever/current smoking, information on IDU (previous/current IDU, IDU partner, use of harm reduction services), HCV coinfection, hepatitis B surface antigen (HBsAg) positivity, STI diagnosis during the most recent pregnancy or postnatally, WHO clinical stage and highly active antiretroviral therapy (HAART). Data linkage allowed women participating in both studies to be identified. Maternal follow-up information was not addressed in this analysis.

The study population for this analysis was 6200 mother–child pairs in the Ukraine ECS (enrolled up to February 2010); 447 women with two infants were included, 45 with three infants and five with four or more infants. Multiple births (*n* = 32) were treated as separate mother–child pairs. By June 2010, 1028 women were being followed in the postnatal cohort.

### Definitions

IDU history (current or past) was assigned according to maternal self-report and/or clinical report; six women without self- or clinical report of IDU delivered infants with NAS and were included in the IDU group. Women were defined as ‘current IDUs’ if IDU was reported during the current pregnancy and/or if their infant had NAS, but we considered all women with an IDU history to be IDUs in recognition that IDU can be a chronic and relapsing condition [31]. In some analyses, women were classified as never IDUs (no IDU history), past IDUs (IDU history but not current) and current IDU. Women with HIV treatment indications were defined as those with CD4 counts <200 cells/mm^3^ and/or with WHO stages 3/4 symptoms. PTD was defined as occurring before 37 completed weeks of gestation. Maternal severe or advanced HIV symptoms were defined as those in WHO clinical stages 3 or 4. Elective caesarean section (CS) deliveries were defined as CS deliveries occurring pre-labour and before rupture of membranes. Time period was classified as follows: 2000–01, 2002–03, 2004–05, 2006–07 and 2008–10. Infants with persistence of antibody beyond 18 months of age and/or a positive virological marker of infection regardless of age were included as HIV-infected. If a child was HIV antibody-negative and no virus had been detected (s)he was classified as uninfected, regardless of age.

### Data analysis

Univariable comparisons were assessed with the χ^2^ test for categorical variables. Logistic regression was used to obtain unadjusted odd ratios (OR), adjusted odds ratios (AOR) and 95% confidence intervals (CI) in separate analyses identifying factors associated with non-receipt of PMTCT prophylaxis, adverse pregnancy outcomes and MTCT. For the MTCT analysis, available variables known to be associated with MTCT risk were included a priori (mode of delivery, preterm delivery, receipt of PMTCT prophylaxis/treatment) [24,32–36]. For the other models, all variables univariably significant (*P* < 0.05) were considered in the multivariable models and retained based on Akaike's information criterion. Statistical analyses were performed with SAS (version 8.02; SAS Institute, Cary, NC, USA).

## RESULTS

Of the 6200 women, 1111 (18%) reported current or previous IDU, of whom 257 (23%) reported or showed evidence of IDU in pregnancy; 192 (19%) of the 1028 women being followed postnatally reported an IDU history. The proportion of IDUs enrolling declined significantly, from 36% (88 of 245) in 2000/01 to 29% (152 of 530) in 2002/03, 24% (230 of 978) in 2004/05, 17% (347 of 2055) in 2006/07 and 13% (294 of 2002) in 2008/10 (χ^2^ = 156.6, *P* < 0.01).

### Maternal characteristics, timing of HIV diagnosis and coinfections

All women were white, and 99% (6159) had been born in Ukraine. Maternal socio-demographic characteristics are presented in Table 1, comparing women with and without an IDU history. IDUs were older than non-IDUs and more likely to report IDU sexual partners, previous pregnancy and more than one pregnancy termination (Table 1). IDUs were less likely to have planned their most recent pregnancy or to have received ANC compared with non-IDUs (Table 1). High levels of cigarette smoking were reported by IDU (Table 1), who reported smoking a median of 20 cigarettes per day (range 3, 30) compared with 10 (range 2, 25) among non-IDUs.

**Table 1:** Socio-demographic characteristics of human immunodeficiency virus (HIV)-infected women with and without injecting drug use (IDU) history

	*IDU*	*Non-IDU*	χ*^2^*
		
	*n (%) or median (IQR)*		*P-value*
		
*ECS cohort (n* = *6104)*^a^	*n* = *1111*	*n* = *4993*	
Age at delivery (years)	28.1 (24.6, 31.8)	25.5 (22.4, 29.5)	*P* < 0.001
Parity (*n* = 6084)			
0	559 (50.7)	3016 (60.5)	42.1
1	383 (34.7)	1477 (29.7)	*P* < 0.001
≥2	161 (14.6)	488 (9.8)	
Previous pregnancy termination(s) (*n* = 6086)			
0	547 (49.5)	3108 (62.4)	72.7
1	259 (23.4)	1000 (20.1)	*P* < 0.001
≥2	299 (27.1)	873 (17.5)	
Marital status (*n* = 6095)			
Married/cohabiting	792 (71.4)	4200 (84.2)	99.4
Single/divorced/widowed	317 (28.6)	786 (15.8)	*P* < 0.001
Ever had an IDU sex partner (*n* = 6065)			
Yes	595 (54.5)	1261 (25.4)	356.4
No	497 (45.5)	3712 (74.6)	*P* < 0.001
Age when leaving full-time education (*n* = 3235)			
≤16 years	204 (36.6)	448 (16.7)	122.5
17–18 years	168 (30.1)	844 (31.5)	*P* < 0.001
≥19 years	186 (33.3)	1385 (51.7)	

*Postnatal cohort (n* = *1028)*	*n* = *192*	*n* = *836*	
History of imprisonment (*n* = 960)			
Yes	38 (20.4)	3 (0.4)	147.2
No	138 (79.6)	771 (99.6)	*P* < 0.001
Current smoking (*n* = 986)			
Yes	178 (93.7)	317 (39.8)	177.8
No	12 (6.3)	479 (60.2)	*P* < 0.001
Previous pregnancy planned (*n* = 996)			
Yes	101 (53.2)	600 (74.4)	33.4
No	89 (46.8)	206 (25.6)	*P* < 0.001
Receipt of ANC in most recent pregnancy (*n* = 1001)			
Yes	159 (82.8)	785 (97.0)	58.4
No	33 (17.2)	24 (3.0)	*P* < 0.001
HIV status of current partner (*n* = 883)			
HIV-positive	82 (50.0)	276 (38.3)	7.6
HIV-negative	33 (20.1)	189 (26.3)	*P* = 0.022
Unknown	49 (29.9)	254 (35.3)	
Living situation (*n* = 975)			
Owned house/apartment	136 (71.6)	568 (72.3)	0.4
Parental home	33 (17.4)	123 (15.7)	*P* = 0.82
Other	21 (11.0)	94 (12.0)	

a 96 women missing data on IDU status. ANC: antenatal care; ECS: European Collaborative Study; IQR: interquartile range.

HIV diagnosis before pregnancy was more common among IDUs than non-IDUs (Table 2), with a significant increase over time in proportion of IDUs aware of their positive HIV status at conception, from 31% (25 of 80) in 2000/01 to 40% (91 of 230) in 2003/04 and 60% (177 of 293) in 2008/09 (χ^2^ = 41.0, *P* < 0.01). Among women with unknown HIV status at conception, 20% (116 of 587) of IDUs were diagnosed through intrapartum testing versus 4% (153 of 3617) of non-IDUs (χ^2^ = 203.4, *P* < 0.01). IDUs had considerably worse HIV clinical and immunological status than non-IDU with 14% having severe/advanced HIV symptoms versus 6% of non-IDUs. (Table 2).

**Table 2:** Maternal human immunodeficiency virus (HIV)-related characteristics and prevention of mother-to-child transmission (PMTCT) use, stratified by injecting drug use (IDU) status (*n* = 6104)

	*IDU*	*Non-IDU*	χ*^2^*
			
	*n* = *1111*	*n* = *4993*	*P value*
		
	*n (%) or median (IQR)*		
Timing of HIV diagnosis (*n* = 6200)			
Pre-pregnancy	524 (47.2)	1376 (27.6)	357.9
1st/2nd trimester	298 (26.8)	2674 (53.6)	*P* < 0.001
3rd trimester	173 (15.6)	790 (15.8)	
Intrapartum	116 (10.4)	153 (3.1)	
WHO clinical stage (*n* = 5303)			
I or II	840 (85.7)	4046 (93.6)	67.4
III or IV	140 (14.3)	277 (6.4)	*P* < 0.001
CD4 count^a^ (*n* = 2401)			
<200 cells/mm^3^	52 (14.4)	163 (7.0)	19.1
200–349 cells/mm^3^	80 (22.2)	439 (21.5)	*P* < 0.001
350–499 cells/mm^3^	104 (28.9)	573 (28.1)	
≥500 cells/mm^3^	124 (34.4)	866 (42.4)	
Median	428 (267, 588)	459 (326, 618)	
PMTCT prophylaxis—mother			
None	136 (12.2)	236 (4.7)	390.4
sdNVP only	295 (26.6)	470 (9.4)	*P* < 0.001
ZDV and sdNVP	275 (24.8)	2232 (44.7)	
ZDV	223 (20.1)	1158 (23.2)	
HAART	182 (16.4)	897 (18.0)	

a First measurement in pregnancy. IQR: interquartile range; HAART: highly active antiretroviral therapy; sdNVP: single-dose nevirapine; ZDV: zidovudine; WHO: World Health Organization.

**Figure 1:**
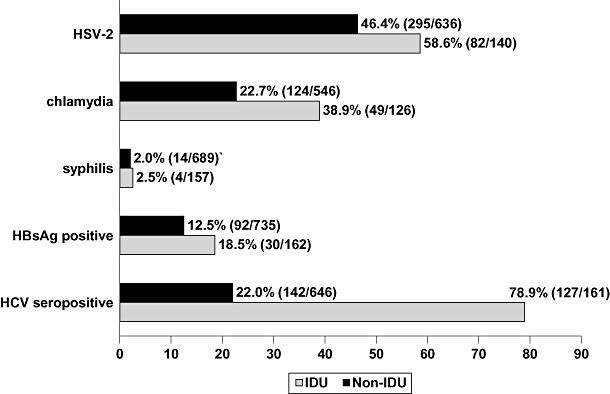
Prevalence of coinfections in injecting drug user (IDU) and non-IDU women (substudy only)

There was a higher prevalence of HCV seropositivity (χ^2^ = 187.5, *P* < 0.001), HBsAg seropositivity (χ^2^ = 6.8, *P* = 0.009), chlamydia (χ^2^ = 14.0, *P* < 0.001) and HSV-2 (χ^2^ = 6.8, *P* = 0.009) among IDU compared with non-IDU, although there was no significant difference with respect to syphilis (Fisher's exact test, *P* = 0.44) (Fig. 1, postnatal cohort only). Of note, 23% of women in the postnatal cohort not reporting IDU were HCV-seropositive.

Home-made opiates were the predominant injected drug, used by 99% (190 of 192) of the IDUs in the postnatal cohort. Overall, 13% (25 of 192) of IDU reported injecting drugs postnatally and 60% (74 of 123) reported having a sexual partner currently injecting drugs. Of the 167 women reporting previous but not current IDU, 157 provided the year of most recent use, with a median of 5 years (range 1–19 years) since last use.

### PMTCT prophylaxis and antiretroviral therapy

Overall, substantially more IDUs received no antenatal or intrapartum PMTCT prophylaxis or only single-dose nevirapine (sdNVP) (Table 2). IDU was associated significantly with non-receipt of antenatal/intrapartum PMTCT prophylaxis (OR 2.81; 95% CI: 2.23, 3.51; *P* < 0.001 compared with non-IDU). In a logistic regression analysis of non-receipt of prophylaxis, current IDU was associated with a more than fourfold increased odds of non-receipt compared with women with no IDU history; this declined to 2.75 increased odds after adjusting for confounding factors (Table 3).

**Table 3:** Factors associated with non-receipt of prevention of mother-to-child transmission (PMTCT) prophylaxis

	*Odds ratio (95% CI)*	*Adjusted odds ratio (aOR) (95% CI), P-value*
IDU history		
Never	1.00	1.00
Past IDU	2.29 (1.76, 2.97)	1.97 (1.51, 2.58) *P* < 0.001*
Current IDU	4.81 (3.43, 6.75)	2.75 (1.90, 3.99) *P* < 0.001*
Late diagnosis of HIV		
No	1.00	1.00
Yes	2.92 (2.34, 3.63)	2.37 (1.88, 3.00) *P* < 0.001
Preterm delivery		
No	1.00	1.00
Yes	3.11 (2.39, 4.06)	2.56 (1.95, 3.38) *P* < 0.001
No cohabiting partner or spouse		
No	1.00	1.00
Yes	2.31 (1.84, 2.91)	1.89 (1.44, 2.33) *P* < 0.001
Time-period		
2000–01	1.00	1.00
2002–03	0.27 (0.16, 0.46)	0.27 (0.16, 0.47) *P* < 0.001
2004–05	0.35 (0.23, 0.54)	0.42 (0.27, 0.67) *P* < 0.001
2006–07	0.32 (0.21, 0.47)	0.44 (0.29, 0.67) *P* < 0.001
2008–10	0.23 (0.16, 0.35)	0.37 (0.24, 0.56) *P* < 0.001

95% CI: 95% confidence interval

* aOR for past versus current injecting drug use (IDU) was 0.72 (0.48, 1.08), *P* = 0.11. HIV: human immunodeficiency virus.

There were 557 women with indications for antiretroviral treatment for their own health; of note, CD4 counts were available only for 2401 women, reflecting limited laboratory capacity, particularly earlier in the study. Of these 557 women, 388 (70%) were non-IDUs and 169 (30%) were IDUs; significantly fewer IDUs received antenatal HAART compared with non-IDUs, 58% (98 of 169) versus 68% (262 of 388) (χ^2^ = 4.68, *P* = 0.031), and the difference remained significant when restricting the analysis to exclude women diagnosed as HIV-infected in the third trimester or at delivery (90 of 147 versus 244 of 347, χ^2^ = 3.98, *P* = 0.049).

### Delivery and infant outcomes

Overall, 52% women delivered vaginally (3856 of 6089). The elective CS rate was 24% (263 of 1111) among IDUs and 34% (1714 of 4991) among non-IDUs (χ^2^ = 47.2, *P* < 0.001). IDUs had higher rates of PTD and LBW infants compared with non-IDUs, with respective PTD rates of 16% (174 of 1110) and 7% (368 of 4985) (χ^2^ = 77.5, *P* < 0.001) and LBW rates of 22% (245 of 1110) and 10% (521 of 4987) (χ^2^ = 111.7, *P* < 0.001). Median head circumference at birth for infants of IDUs was 33 cm [interquartile range (IQR) 32, 34] and 34 cm [33,35] for other infants. Current IDUs had rates of PTD and LBW infants of 22% (56 of 257) and 31% (79 of 257), respectively; 159 (62%) of current IDUs had infants with NAS, this included six women with no clinical or self-report of IDU at enrolment and 38 women reporting former IDU, all of whom were classifed as current IDU according to our study definitions.

Overall, 25% (275 of 1110) IDUs had a PTD and/or a LBW infant (‘adverse pregnancy outcome’). IDU was associated significantly with adverse pregnancy outcomes in univariable logistic regression analyses, together with late HIV diagnosis (used as a proxy for no or inadequate ANC), having a cohabiting partner/spouse and PMTCT prophylaxis (*P* < 0.05); there were no significant trends in adverse pregnancy outcomes over calendar time (overall and among IDUs). The multivariable model (*n* = 6040), which included all the above factors, indicated that the adjusted odds of an IDU having an adverse pregnancy outcome compared with a non-IDU was 1.84 (95% CI: 1.55, 2.18, *P* < 0.001). Repeating this analysis, with IDU re-classified, past and current IDU were associated with the outcome [respective AORs 1.67 (1.38–2.03) and 2.38 (1.78–3.9) versus never IDU]; the AOR for past versus current IDU was 0.70 (95% CI: 0.51, 0.97, *P* = 0.03).

### MTCT

Overall, <1% (*n* = 61) women breastfed their infants, eight of whom were IDUs. Unadjusted MTCT rates were 5.9% (205 of 3489, 95% CI: 5.1–6.7) for non-IDUs and 10.8% for IDUs: 10.0% for past IDUs (63 of 629, 95% CI: 7.8–12.6), 13.1% (29 of 221, 95% CI: 9.0–18.3) for current IDUs and 21.8% (19 of 87, 95% CI: 13.7–32.0) for IDUs with intrapartum HIV diagnosis. MTCT rates declined over calendar time among IDUs, from 17.6% (13 of 74, 95% CI: 9.7–28.1) in 2000–01 to 3.8% (six of 154, 95% CI: 1.4–8.3) in 2008–09 and among non-IDUs from 11.6% (17 of 146, 95% CI: 6.9–18.0) to 2.8% (26 of 933, 95% CI: 1.8–4.1) in the same years. In unadjusted logistic regression, IDU was associated with a nearly twofold increased risk of MTCT (*P* < 0.001) (Table 4). Classifying IDU as current, past and never, the ORs of MTCT for past IDU and for current IDU versus never were 1.75 (95% CI: 1.30, 2.36) and 2.43 (1.60, 3.68), respectively. In multivariable analysis including IDU as a dichotomous variable (*n* = 4328), IDUs had a 32% increased risk of transmitting infection to their infants compared with non-IDU (aOR 1.32) (Table 4). Repeating this analysis, adjusting for time-period, the AOR for IDU decreased slightly to 1.28 (95% CI: 0.97, 1.70), associated with MTCT with borderline statistical significance (*P* = 0.08).

**Table 4:** Risk factors associated with mother-to-child transmission (MTCT) of human immunodeficiency virus (HIV)

	*MTCT rate (%)*	*Odds ratio (95% CI)*	*Adjusted odds ratio (95% CI), P-value*
IDU history			
No	205/3485 (5.9%)	1.00	1.00
Yes	91/843 (10.8%)	1.94 (1.49–2.51)	1.32 (1.00–1.75) *P* = 0.049
Premature delivery			
No	247/3976 (6.2%)	1.00	1.00
Yes	49/352 (13.9%)	2.44 (1.76–3.39)	1.61 (0.13–2.29) *P* = 0.08
Mode of delivery			
Vaginal	223/2676 (8.3%)	1.00	1.00
Emergency CS	10/190 (5.3%)	0.61 (0.32–1.17)	0.69 (0.35–1.34) *P* = 0.28
Elective CS	63/1462 (4.3%)	0.50 (0.37–0.66)	0.71 (0.18–0.96) *P* = 0.03
AN/IP PMTCT prophylaxis			
None	48/220 (21.8%)	1.00	1.00
sdNVP	81/571 (14.2%)	0.59 (0.40–0.88)	0.61 (0.35–0.91) *P* = 0.01
ZDV	75/1761 (4.3%)	0.16 (0.11–0.24)	0.21 (0.14–0.31) *P* < 0.001
ZDV with sdNVP	85/1193 (7.1%)	0.28 (0.19–0.41)	0.36 (0.24–0.54) *P* < 0.001
HAART	7/583 (1.2%)	0.04 (0.02–0.10)	0.06 (0.02–0.13) *P* < 0.001

AN: antenatal; IP: intrapartum; 95% CI: 95% confidence interval; CS: caesarean section; IDU: injecting drug use; HAART: highly active antiretroviral therapy; PMTCT: prevention of mother-to-child transmission; sdNVP: single-dose nevirapine; ZDV: zidovudine.

## DISCUSSION

In this Ukrainian cohort, spanning more than 10 years, just under one-fifth of women were current or past-IDUs. IDUs had higher prevalence of coinfections, advanced HIV disease and severe immunosuppression compared with other women. One in 10 IDUs did not access PMTCT prophylaxis, mainly because they were diagnosed too late. Adverse pregnancy outcomes, including PTD and LBW as well as MTCT, were more frequent in IDUs and IDU history was associated independently with a 30% increased MTCT risk.

IDUs are frequently socially marginalized and socio-economically deprived, and can be hard to reach with services, including HIV testing. Coverage of IDUs with HIV testing (in previous 12 months) is an estimated 30% in Ukraine [37]. More IDUs here knew their HIV-positive status before pregnancy than non-IDUs (47% versus 28%), probably reflecting HIV testing within addiction services. However, among women with unknown HIV status at conception, IDUs were more likely to be diagnosed late. One in 10 IDUs were diagnosed intrapartum, with sdNVP the only potential option for PMTCT prophylaxis compared with 3% of other women, reflecting the fact that one in six received no ANC. In a study in St Petersburg, Russian Federation, two-thirds of women presenting in labour with unknown HIV status were IDUs, mostly without ANC [27]; here the equivalent figure was 43%. IDU has been identified previously as a risk factor for non-receipt of PMTCT prophylaxis in western Europe [25,26], but not consistently [38].

In unadjusted analyses, IDU was associated with a twofold increased MTCT risk and IDUs contributed 31% of all vertical transmissions. The main mechanisms behind the elevated MTCT risk associated with IDUs were most probably their lower coverage with PMTCT interventions and higher rate of PTD. Infants of current IDUs were at greatest risk of MTCT, with a rate of 13.1%. Such infants were significantly more likely to be delivered preterm than other infants and had a nearly threefold increased probability of non-receipt of PMTCT prophylaxis, resulting in a nearly 2.5-times increased transmission risk versus never IDUs in unadjusted analyses. The finding that current IDUs were at greatest risk of MTCT underscores the need to strengthen harm reduction in Ukraine as a key component of the broad PMTCT strategy. IDUs remained associated significantly with a 30% increased MTCT risk in adjusted analysis; this might be explained by factors including poorer adherence to prophylaxis/treatment and higher rates of some coinfections, including HCV [39–43], but we were unable to explore further due to limited data.

The trend of declining MTCT rates over time applied to IDUs as well as other women, with both groups having rates <4% by 2008–09. This is above the target for ‘virtual elimination’ of HIV among infants [44], but is very encouraging and largely reflects the introduction of HAART for PMTCT. Challenges remain, however: although non-receipt of PMTCT prophylaxis declined significantly over time due to scaling-up of the PMTCT programme in Ukraine, IDUs still had a two- to threefold increased risk of non-receipt after adjustment for time-period and other confounding factors, including late HIV diagnosis and PTD.

NAS, which occurs in approximately 60% of all newborns exposed *in utero* to opiates [45], was identified in 62% of newborns of current IDUs. Although NAS is easy to treat with morphine drops, this treatment is not currently available in Ukraine. Of particular public health concern is that nearly a third of PTD here were contributed by IDUs, probably reflecting factors including no or limited ANC, poor nutrition, alcohol use and smoking, maternal infections, socio-economic factors and direct effect of illicit drugs [18,31,46,47]. Smoking among IDUs was nearly universal (94%), and is associated with more intense NAS [48]. Maternal drug use is associated with infant abandonment in eastern Europe [49–51], and we reported recently that infants with NAS in our cohort were 10 times more likely to be abandoned than other infants [52].

Problems faced by IDUs in accessing addiction, HIV, reproductive and other services [18,31,53] reflect barriers to service access (including geographical or administrative barriers and chaotic and/or mobile life-styles), but may also arise from specific avoidance of services following prior negative/stigmatizing experiences [31,54,55]. Our study population most probably faced a double stigma, due to their IDU and HIV status, which may have been compounded by their gender and pregnancy [56]. The higher rate of pregnancy terminations and of unplanned pregnancy in IDUs compared with non-IDUs are consistent with inadequate access to services. Although the proportions of IDUs and non-IDUs receiving antenatal HAART were similar, IDUs had worse health status, and among women with treatment indications IDUs were significantly less likely to receive HAART. This inequity is consistent with other findings, demonstrating that IDUs are less likely to receive HAART or start HAART later than non-IDUs [42,53,55,57].

A comprehensive package of care for IDUs should include HIV testing, treatment and care, tuberculosis (TB) and STI services and harm reduction, including needle/syringe exchange programmes (NSP) and opioid agonist maintenance treatment [58]. For female IDUs, linkages between these services and reproductive health services, including pregnancy testing, contraception and PMTCT are very important [58], but frequently weak. Provision of multi-disciplinary care is particularly challenging in Ukraine, where there remains a traditional vertical health system with few functional linkages between services. Coverage with NSP was initially low in Ukraine, with fewer than 10% of IDUs estimated to be reached by early 2003 [59]; however, recent estimates indicate that 32% of IDUs were reached by preventive interventions in the previous 12 months in 2008 [27].

Implementation of opioid agonist maintenance treatment for IDUs has also been slow, with methadone maintenance not available until mid-2008 [7]; no women in our postnatal study received methadone (no data in the ECS). WHO guidelines include a strong recommendation for provision of such treatment to pregnant IDUs [58], and the first pilot study of methadone maintenance in pregnant women will soon start in Ukraine. An additional benefit of a daily intake of methadone is the facilitation of close medical monitoring in pregnant drug addicts.

One of the most effective ways of preventing HIV infection in infants is to prevent their mothers from becoming infected in the first place. Recent models suggest that HIV prevalence in Odessa could be reduced by 41% over the next 5 years if there were a 60% reduction in unmet need for services, including NSP, opioid agonist maintenance treatment and antiretroviral therapy started promptly when indicated [60]. The specific barriers that female IDUs face in accessing harm reduction services and the role these play in their increased risk of HIV acquisition require further investigation. Prevention of unintended pregnancies in HIV-infected women is another important approach to preventing infant infections. Nearly half the IDUs here had not planned their pregnancy, significantly more than other women, in a context of low levels of effective family planning use and identified considerable unmet need for contraception [30].

This study is limited by its observational nature and the potential for confounding. Social desirability bias may have prevented some women from reporting IDU. Although our classification also used clinical observation and NAS, up to 40% of infants with fetal exposure to opiates do not develop NAS. We therefore cannot exclude the possibility that some IDUs may have been included in the non-IDU group, underscored by the 22% prevalence of HCV in the non-IDU group. Our study population lives in cities with the highest antenatal HIV prevalence in Ukraine, including Odessa, Kyiv and Mykolaiv [9], and we estimate that approximately 30% of HIV-infected women delivering in Ukraine are included in our study (1166 of 3649 in 2008) [10]. Our study excluded women terminating their pregnancy and stillbirths; such groups may include more IDUs than the study population.

Some important successes documented here include the increasing proportion of IDUs knowing their HIV status before pregnancy, the declining proportion receiving no PMTCT prophylaxis and substantially lower MTCT risk in recent years, regardless of IDU status. However, important challenges remain, such as provision of comprehensive care to female IDUs, including harm reduction, family planning and HIV treatment as well as provision of ANC and PMTCT, with an emphasis on improving timely access.

## Declarations of interest

Claire Thorne holds a Wellcome Trust Research Career Development Fellowship, which supports the Ukraine Cohort Study of HIV-positive Childbearing Women. The ECS has previously received funding from the European Union Sixth Framework Programme (grant agreement PENTA/ECS 018865) and the research leading to these results has received funding from the European Union Seventh Framework Programme (FP7/2007–13) under EuroCoord grant agreement no. 260694. Some of this work was undertaken at GOSH/UCL Institute of Child Health which received a proportion of funding from the UK Department of Health's NIHR Biomedical Research Centres funding scheme. The Centre for Paediatric Epidemiology and Biostatistics also benefits from funding support from the Medical Research Council in its capacity as the MRC Centre of Epidemiology for Child Health. Claire Thorne has previously carried out consultancy work for pharmaceutical industry (Roche).
